# miR395e from *Manihot esculenta* Decreases Expression of PD-L1 in Renal Cancer: A Preliminary Study

**DOI:** 10.3390/genes16030293

**Published:** 2025-02-27

**Authors:** Joanna Bogusławska, Aizhan Rakhmetullina, Małgorzata Grzanka, Alex Białas, Beata Rybicka, Joanna Życka-Krzesińska, Tomasz Molcan, Piotr Zielenkiewicz, Leszek Pączek, Agnieszka Piekiełko-Witkowska

**Affiliations:** 1 Department of Biochemistry and Molecular Biology, Centre of Translational Research, Centre of Postgraduate Medical Education, ul. Marymoncka 99/103, 01-813 Warsaw, Poland; joanna.boguslawska@cmkp.edu.pl (J.B.); malgorzata.grzanka@cmkp.edu.pl (M.G.); alex.bialas@cmkp.edu.pl (A.B.); beata.rybicka@cmkp.edu.pl (B.R.); joanna.zycka-krzesinska@cmkp.edu.pl (J.Ż.-K.); 2 Department of Bioinformatics, Institute of Biochemistry and Biophysics, Polish Academy of Sciences, ul. Pawińskiego 5a, 02-106 Warsaw, Poland; arakhmet@ibb.waw.pl (A.R.); t.molcan@pan.olsztyn.pl (T.M.); piotr@ibb.waw.pl (P.Z.); leszek.paczek@wum.edu.pl (L.P.); 3 Molecular Biology Laboratory, Institute of Animal Reproduction and Food Research, Polish Academy of Sciences, Tuwima Street 10, 10-748 Olsztyn, Poland; 4 Department of Systems Biology, Institute of Experimental Plant Biology and Biotechnology, University of Warsaw, ul. Miecznikowa 1, 02-096 Warsaw, Poland; 5 Department of Clinical Immunology, Medical University of Warsaw, 02-006 Warsaw, Poland

**Keywords:** renal cell cancer, RCC, PD-L1, mes-miR395e, plant miRNA, immune checkpoint inhibitor, cassava, *Manihot esculenta*, horizontal miRNA transfer

## Abstract

**Background/Objectives**: microRNAs are small non-coding RNAs that regulate gene expression by inducing mRNA degradation or inhibiting translation. A growing body of evidence suggests that miRNAs may be utilized as anti-cancer therapeutics by targeting expression of key genes involved in cancerous transformation and progression. Renal cell cancer (RCC) is the most common kidney malignancy. The most efficient RCC treatments involve blockers of immune checkpoints, including antibodies targeting PD-L1 (Programmed Death Ligand 1). Interestingly, recent studies revealed the cross-kingdom horizontal transfer of plant miRNAs into mammalian cells, contributing to the modulation of gene expression by food ingestion. Here, we hypothesized that PD-L1 expression may be modulated by miRNAs originating from edible plants. **Methods**: To verify this hypothesis, we performed bioinformatic analysis to identify mes-miR395e from *Manihot esculenta* (cassava) as a promising candidate miRNA that could target PD-L1. To verify PD-L1 regulation mediated by the predicted plant miRNA, synthetic mes-miR395 mimics were transfected into cell lines derived from RCC tumors, followed by evaluation of PD-L1 expression using qPCR and Western blot. **Results**: Transfection of mes-miR395e mimics into RCC-derived cell lines confirmed that this miRNA decreases expression of PD-L1 in RCC cells at both mRNA and protein levels. **Conclusions**: This preliminary study shows the promise of plant miRNA as potential adjuvants supporting RCC treatment.

## 1. Introduction

Immune checkpoint inhibitors involving antibodies that block PD-1/PDL-1 interactions have revolutionized the treatment of cancer. PD-1 (programmed cell death receptor-1; alias CD279) is expressed at the surface of T and B lymphocytes as well as myeloid cells. PD-L1, the primary ligand of PD-1, is a glycoprotein localized in the plasma membrane of antigen-presenting cells (APCs). Binding of PD-L1 to PD-1 triggers signaling pathways that prevent T cell activation and downregulation of the immune response, providing an important homeostatic control of the immune system [1]. Cancer cells avoid immune surveillance by overexpressing PD-L1 and switching off the T cell response. Antibodies that target PD-1 (e.g., nivolumab) or PD-L (atezolizumab) prevent PD-1/PD-L1 interaction, relieving the blockade of the immune response towards tumor cells, with significant clinical benefit to patients [1,2].

Renal Cell Cancer (RCC) is the most common type of kidney cancer. Each year, approximately 300,000 patients are diagnosed with RCC worldwide. Of these patients, 20–30% already have metastatic disease at the time of diagnosis, which significantly affects their prognosis. A subset of cancers of unknown primary (CUP) presents the histological and immunohistochemical features of RCC and may contribute to the increasing RCC incidence [3,4]. Recent advancements in renal cell carcinoma (RCC) diagnostics such as enhanced imaging techniques and incorporation of artificial intelligence (AI) have improved early detection and characterization of tumors [5,6]. Regarding the molecular RCC diagnostics, great hopes are being placed on the use of exosomes. These small vesicles (~30 to ~150 nm in diameter) are derived from the intracellular multivesicular bodies (MVBs) and are released into the extracellular space. The cargo in tumor-derived exosomes (e.g., proteins, metabolites, non-coding RNAs, in particular miRNAs) can be detected in a patient’s serum/plasma, urine or saliva, offering valuable potential biomarker targets for early detection and monitoring of the disease [7]. While the primary RCC can be cured effectively by surgical resection, metastatic RCC (mRCC) is treatment-resistant, and patients often relapse. Therapies based on the blockade of PD-1/PD-L1 interaction are currently considered as the most efficient therapy for mRCC. However, up to 75% of RCC patients still remain refractory to antibodies targeting PD-1/PD-L1 [2]. Furthermore, tyrosine kinase inhibitor (TKI) monotherapy is a suitable first-line therapy for many patients ineligible for immunotherapy, as supported by the STAR trial results [8]. Heavily pre-treated relapsed patients who have previously received immune checkpoint and antiangiogenic therapies can be offered belzutifan, a small-molecule inhibitor of HIF-2α, which has recently demonstrated significant benefits over everolimus in terms of progression-free survival and objective response in participants with advanced clear-cell RCC [9]. Thus, there is an urgent need for identification of novel treatments that could boost anti-cancer immune responses.

MicroRNAs (miRNAs) are small (18–22 nt) non-coding RNAs that regulate gene expression by binding to mRNA and targeting them for degradation or inhibiting translation. The role of microRNAs in cancers is well recognized. miRNAs can target oncogenes and tumor suppressors, thereby contributing to cancer initiation, progression, and response to therapy [10]. Recent studies provide evidence on the cross-kingdom horizontal miRNA transfer, highlighting the potential of plant-derived miRNAs in cancer therapy and offering a novel approach to target human cancer cells [11,12,13]. Plant miRNAs can enter human cells through dietary intake and exert regulatory effects on gene expression. After binding to complementary sequences in the 3′ untranslated regions (UTRs) of target mRNAs, exogenous plant miRNAs can trigger mRNA degradation or translational repression. This process is facilitated by the RNA-induced silencing complex (RISC), which incorporates the miRNA and guides it to its target mRNA. Several plant miRNAs have shown promise in preclinical studies for their anti-cancer properties. For instance, miR-159 from *Arabidopsis thaliana* has been demonstrated to target and downregulate the expression of TCF7, a transcription factor involved in the Wnt signaling pathway. By inhibiting TCF7, miR-159 can suppress cancer cell proliferation and attenuate the growth of breast cancer xenografts in mice [14]. Soybean gma-miR159a inhibits the proliferation of colorectal cancer Caco-2 cells and targets TCF7 expression [15]. mtr-miR-5754 and gma-miR4995 directly target the tumor-associated long non-coding RNA MALAT1 and NEAT1 and attenuate proliferation of colorectal cancer cells [16].

To our knowledge, while there are numerous studies showing that extracts from medicinal plants can inhibit PD-L1 (e.g., [17,18,19]), none of them have explored the potential of plant miRNAs to target PD-L1 expression. Therefore, here we searched plant miRNAs to find one that could target PD-L1 and verified its efficacy in renal cancer cells.

## 2. Materials and Methods

Bioinformatic analysis: The nucleotide sequence of the PD-L1 mRNA (NM_014143.4) was obtained from NCBI GenBank (http://www.ncbi.nlm.nih.gov, accessed on 13 May 2022), with the longest transcript (NM_014143.4) chosen for analysis, while the plant miRNA sequences were retrieved from miRBase v.22 (http://www.mirbase.org/, accessed on 13 May 2022). To identify plant miRNAs that may interact with human PD-L1 mRNA, a search for potential miRNA candidates was conducted using psRNATarget [20]. miRNAs from all available species were selected as input. The psRNATarget program characterizes plant miRNA binding to mRNA target transcripts by evaluating the complementary interaction between miRNAs and their targets using a predefined scoring system, and by determining target site accessibility through the calculation of unpaired energy. An improved algorithm was applied for miRNA candidate identification: penalties were assigned as 0.5 for extending gaps, 2 for opening gaps, 0.5 for G.U pairs, and 1 for other mismatches. The High Scoring Pair size was set to 19, with the seed region defined as nucleotides 2–13, and the minimum expectation score was set to 5.0.

Next, to conduct the cross-kingdom search for plant miRNAs targeting the mRNA of the PD-L1 gene, the Tools4Mirs platform was utilized [21]. This tool was used to validate the miRNAs identified by psRNATarget as potential regulators of the human PD-L1 gene mRNA. Tools4miRs offers a web-based miRNA target prediction meta-server that integrates multiple target prediction tools, including miRanda [22], PITA [23], PsRobot [24], RNA22 [25], RNAhybrid [26], Guugle [27], TargetSpy [28], and miRmap [29] into its analysis.

Cell culture propagation: Human renal cancer-derived cell lines, including 786-O (CRL-1932; ATCC), Caki-1 (HTB-46; ATCC) and KIJ265T (a kind gift of Doctor John A. Copland and Mayo Foundation of Medical Education and Research), were cultured as earlier described [30]. Briefly, and in line with provider’s instructions, the 786-O and Caki-1 cell lines were cultured in RPMI-1640 and McCoy’s 5a, respectively, while the KIJ265T cells were propagated in MEM medium supplemented with 1 mM sodium pyruvate and non-essential amino acids. All media were purchased from ThermoFisher Scientific (Waltham, MA, USA). To make the complete growth medium, penicillin–streptomycin (Sigma, St. Louis, MO, USA) and foetal bovine serum (ThermoFisher Scientific) was added to a final concentration of 10%.

Immunocytochemistry: Cells were fixed with 4% PFA at 48 h after seeding on coverslips and then permeabilized using 0.25% Triton X-100. The next step involved blocking nonspecific binding with 2% BSA in TBST, followed by incubation with Phalloidin-Atto 488, solution (Sigma-Aldrich, St. Louis, MO, USA) and then DAPI. The prepared cells were imaged using a LSM 800 confocal laser scanning microscope, Axio Observer Z1 with ZEN 3.7 software (Carl Zeiss AG, Oberkochen, Germany).

miRNA transfection: 5 × 10^4^ cells were seeded in a 12-well plate and transfected with synthetic plant miRNA mimics (synthesized based on the mes-miR385e sequence by Future Synthesis, Poznań, Poland) at final concentrations of 100 pmol or 200 pmol per well using Lipofectamine RNAiMAX (Thermo Fisher Scientific) according to the manufacturer’s protocol. Transfection complexes were prepared by diluting the appropriate amount of synthetic miRNA in Opti-MEM (Gibco) and mixing with RNAiMAX reagent. The mixture was incubated for 5 min at room temperature and then added to the cells. The transfection was carried out for 48 h, after which total RNA was isolated. After 72 h, protein extraction was performed for subsequent analyses.

RNA isolation and qPCR: RNA isolation and qPCR were performed as earlier described [31] using the following primer sequences: PDL1_F: TATGGTGGTGCCGACTACAA, and PDL1_R: TGCTTGTCCAGATGACTTCG. HPRT was used as a reference gene (primers are given in [31]).

Cloning of the miRNA target sites and luciferase assays were performed as described previously [31].

Protein isolation and Western blots: Protein was isolated using RIPA buffer. Western blots were performed using 20 micrograms of protein per SDS-PAGE well. The proteins were transferred onto nitrocellulose (0.45 um) membrane, followed by blocking o/n in 5% non-fat milk. Incubation in primary antibody (Abcam, cat. No. ab213524, dilution 1:1000, Cambridge, UK) was performed at 4 °C o/n. Next, the membranes were incubated in a secondary antibody (Dako, Cat. No. P0448, dilution 1:10,000, Glostrup, Denmark) for 1 h at RT, followed by signal development using SuperSignal™ West Dura Extended Duration Substrate (ThermoFisher Scientific) and exposure to Super RX-N Fuji Medical X-Ray film. Next, the membranes were incubated in anti β-Actin primary antibodies (SantaCruz Biotechnology, Cat. No. sc69879, dilution 1:20,000, Dallas, TX, USA) for 1 h at RT, and in secondary antibodies (Dako, cat. No. P0447, dilution 1:10,000) for 1 h at RT, followed by signal development using SuperSignal™ West Pico PLUS Chemiluminescent Substrate (ThermoFisher Scientific) and film exposure.

## 3. Results

### 3.1. Bioinformatic Analysis Predicts Plant miRNAs That Have the Potential to Regulate PD-L1 Expression

To identify miRNAs potentially interacting with the PD-L1 gene, psRNATarget was initially used. Subsequently, the Tools4Mirs platform was employed to perform a cross-kingdom search for plant miRNAs targeting PD-L1 mRNA and validate the results. During validation, the following criteria were applied to narrow down the large number of predicted miRNAs: An additional search was conducted using six tools—PsRobot, RNA22, GUUGle, RNAhybrid, miRanda, and PITA. The results for RNA22 were restricted to entries with a sufficiently low energy threshold of −19 kcal/mol and a minimal number of gaps or “G-U pairs” within the seed region. For both the RNA22 and GUUGle programs, the minimum number of paired base pairs was set to 12 bp, with an expected seed region size of 7 bp for RNA22. Additionally, the PITA program results were limited to entries with a ΔΔG score of −10. For further analysis, only miRNA–PD-L1 pairs predicted by at least three of the mentioned software tools were included. This approach was used to ensure high-confidence results, which were then regarded as the final predictions. The obtained dataset was analyzed to identify the most relevant plant miRNA targeting human PD-L1.

The miRNA mes-miR395e from *M. esculenta* was chosen for further investigation based on its fulfillment of all criteria, its predicted miRNA–mRNA interaction characteristics across multiple programs, as well as its occurrence in a plant known for its edibility and medicinal properties in humans [32].

**Figure 1:**
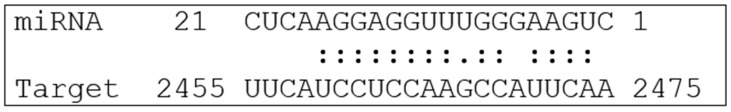
Predicted interaction of mes-miR395e with human PD-L1 mRNA (NM_014143.4).

The scheme of the predicted interaction of mes-miR395e with mRNA of the human PD-L1 gene presented in Figure 1 demonstrates that nucleotides of miRNA form hydrogen bonds in the 3′UTR of mRNA.

These data indicate a strong interaction between the miRNA and PD-L1 mRNA, demonstrating high efficiency. This is due to the complementary interaction of these molecules, facilitated by both canonical and non-canonical nucleotide pairs. Considering non-canonical pairings, such as G–U, enhances the interaction between the miRNA and mRNA, thereby increasing the effectiveness of this interaction [33,34,35].

### 3.2. Mes-miR395e Attenuates PD-L1 Expression in Renal Cancer Cells

**Figure 2:**
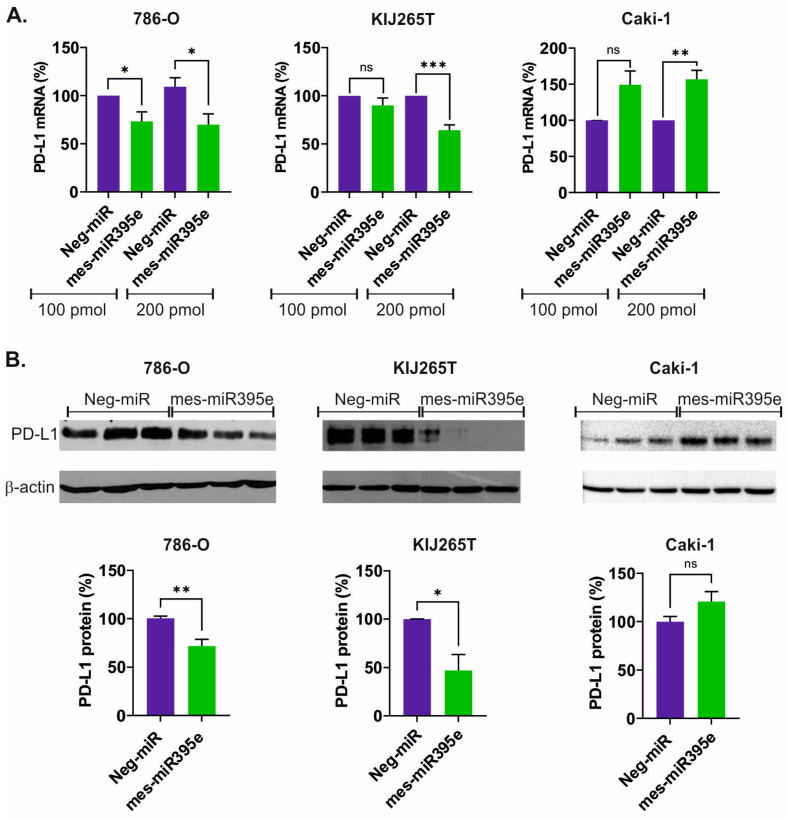
mes-miR395e from *M. esculenta* downregulates PD-L1 in renal cancer cells. (**A**) The plots show results of qPCR analysis of PD-L1 expression in RCC cells transfected with mes-miR395e mimic or non-targeting scrambled control oligonucleotide (Neg-miR). (**B**) Western blot analysis PD-L1 protein expression in RCC cells transfected with mes-miR395e mimic or non-targeting scrambled control oligonucleotide (Neg-miR). Representative blots are shown. All blots that served for densitometric analysis are shown in Supplementary Figure S1. The plots below show results of densitometric scanning of blots. PD-L1 protein expression was normalized using β-actin. Statistical analysis was performed using *t*-test. * *p* < 0.05, ** *p* < 0.01, *** *p* < 0.001, ns: non significant.

To analyze the impact of mes-miR395e on PD-L1 expression, miRNA mimics were transfected into cell lines derived from primary RCC (786-O, KIJ265T) and one cell line derived from RCC skin metastasis (Caki-1). qPCR analysis revealed that mes-miR395e mimics decreased the expression of PD-L1 mRNA in both cell lines derived from primary tumors (Figure 2A). Next, we analyzed the influence of mes-miR395e on PD-L1 protein expression. Western blot analysis confirmed downregulation of PD-L1 protein in cells transfected with mes-miR395e when compared with cells transfected with non-targeting scrambled control oligonucleotide (Figure 2B, Supplementary Figure S1).

Surprisingly, analysis of mes-miR395e effects in the Caki-1 cell line derived from skin metastasis resulted in opposite effects. Specifically, we observed induction of PD-L1 mRNA following transfection of the mes-miR395e mimic (Figure 2A). However, there was no statistically significant effect of mes-miR395e on the expression of PD-L1 protein in the Caki-1 cell line (Figure 2B).

Next, we evaluated if mes-miR395e could directly target PD-L1 mRNA. To this end, we co-transfected mes-miR395e mimics with a luciferase-reported plasmid bearing the predicted mes-miR395e binding site, cloned from the PD-L1 gene sequence. There was no change in luciferase activity following mes-miR395e mimic transfections, suggesting that this miRNA possibly downregulates PD-L1 expression by indirect mechanisms.

Finally, we checked if the morphology of RCC cells was altered following mes-miR395e transfections. We did not observe any significant changes in cell morphology (Supplementary Figure S2).

Altogether, these data indicate that the mes-miR395e mimic could attenuate PD-L1 expression in RCC cell lines derived from primary tumors.

## 4. Discussion

To our knowledge, this is the first study that shows the potential of plant miRNA to regulate the expression of a gene involved in immune checkpoint regulation in cancer. Specifically, we demonstrate that a mimic of mes-miR395e, a plant miRNA derived from *M. esculenta*, can decrease the expression of PD-L1, suggesting its potential utility in immune-based anti-cancer therapy.

**Figure 3:**
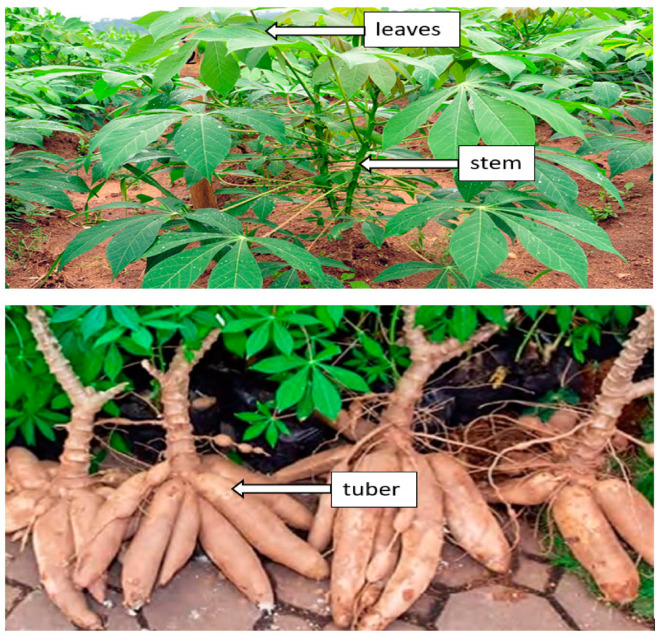
Cassava (*M. esculenta)* plant. (Image sourced from the previously published study [41] that was published as open access article distributed under the terms and conditions of the Creative Commons Attribution (CC BY) license (https://creativecommons.org/licenses/by/4.0/, accessed on 20 February 2025).

*M. esculenta* (Figure 3) is an edible plant and a crucial component of diets in African, Asian, and Latin-American populations [36]. Despite its utility as a source of nutrients, consumption of unprocessed cassava is associated with a risk of poisoning due to the presence of toxic substances including cyanogenic glucosides. Traditional as well as modern processing techniques minimize the content of cyanide from the cassava-based foods [37]. Moreover, high doses (2000 mg/kg) of ethanolic leaf extracts of *M. esculenta* did not produce any adverse effects in rats while protecting against gastric ulceration [38]. Remarkably, anti-cancer properties of cassava extracts have already been demonstrated in experimental studies. For instance, Sreejith et al. showed that cassava extracts attenuate the viability of glioblastoma cells in a dose-dependent manner [39]. These effects were associated with increased generation of ROS and decreasing concentrations of nitrile radicals [39]. In promyelocytic leukemia cells, aqueous cassava extracts attenuated viability and PMA-induced ROS production [36]. Cassava extracts diminished the growth of cell lines derived from breast cancer, colon adenocarcinoma and acute myelogenous leukemia [40]. However, the impact of cassava miRNAs has never been analyzed in the context of cancer.

Although several previous studies showed that plant extracts can modulate the expression of PD-L1 [42,43,44,45], to our knowledge, none of them suggested that these effects could be mediated by miRNAs. This is surprising since plant miRNAs have been widely explored as potential therapeutic tools in the treatment of cancer. For instance, miR159 from *Arabidopsis thaliana* and soybean targeted transcription factor TCF7 in breast cancer to decrease expression of the MYC oncogene and attenuated the growth of breast cancer in vitro and in vivo [14]. A mixture of seven plant miRNAs inhibited proliferation of colorectal cancer cells and suppressed expression of the oncogenic lncRNAs MALAT1 and NEAT1. Detailed analysis revealed that mtr-miR-5754 and gma-miR-4995 directly targeted and decreased expression of both lncRNAs in colon cancer cells [16]. Similar findings were also obtained in the context of kidney cancer. Specifically, lb-miR166a from *Lycium barbarum*, a traditional Chinese medicinal herb, promoted the apoptosis and inhibited the proliferation, invasion and metastasis of renal cancer cells. Moreover, it inhibited the growth of RCC tumors in vivo [46]. Furthermore, plant miRNAs can affect the functioning of human immune cells, including Th2 lymphocytes and dendritic cells, and also have beneficial effects on autoimmune disease in mouse models [13]. All these data clearly support the idea of potential anti-cancer therapies based on plant miRNAs.

Our study has some obvious limitations. Firstly, we did not test the effect of the mes-miR395e mimic on normal proximal tubules. Physiologically, the latter express PD-L1, which protects them against T cell mediated autoimmune attack. PD-L1 expression is dramatically upregulated in proximal tubules in response to inflammation and acute kidney disease. The use of ICIs such as anti-PD-L1 antibodies is associated with immune-related adverse effects (irAEs) resulting from drug-induced activation of the immune system. Acute kidney injury is the most common irAE [47]. Therefore, the impact of mes-miR395e on normal proximal tubules should definitely be evaluated by future experimental studies. The other limitation is that we did not discover the mechanism by which mes-miR395e reduces PD-L1 expression. No change in luciferase activity in the miR-target sequence reporter assay suggests that mes-miR395e does not bind to the predicted binding sequence in 3′UTR of PD-L1 mRNA. MiRNAs can affect gene expression by multiple indirect mechanisms, such as nuclear actions affecting the transcription and epigenetic regulation, or via crosstalk with ubiquitination systems [48,49]. The knowledge of mechanisms by which plant miRNAs influence the expression of human genes is still in its infancy and requires comprehensive and systematic studies. Finally, it should be explored why PD-L1 expression was not changed by the mes-miR395e mimic in all tested cell lines. The lack of PD-L1 expression changes in Caki-1, in contrast to the other two tested RCC cell lines, suggests that the effects of plant miRNAs may depend on the molecular profile of the targeted cell. All these questions should be addressed by future experimental studies.

In conclusion, here, we present data showing that inhibition of PD-L1 expression can be achieved with the support of the plant miRNA mes-miR395e. Future studies are needed to verify the efficacy of mes-miR395e in experimental anti-RCC therapy in vivo.

## Data Availability

The original contributions presented in this study are included in the article/Supplementary Materials. Further inquiries can be directed to the corresponding author.

## Supplementary Materials

The following supporting information can be downloaded at: https://www.mdpi.com/article/10.3390/genes16030293/s1. Supplementary Figure S1: Full data of Western blot analysis of RCC cell lines transfected with mes-miR395e mimic or non-targeting scrambled control oligonucleotide. Supplementary Figure S2: Representative confocal microscopy images of 786-O cells transfected with non-targeting scrambled control oligonucleotide (Neg-miR) or mes-miR395e mimic.
