# Supplementary material for: miR395e from Manihot esculenta Decreases Expression of PD-L1 in Renal Cancer: A Preliminary Study
Source: Genes (Basel). 2025 Feb 27;16(3):293. doi: 10.3390/genes16030293 (PMC11942022; doi:10.3390/genes16030293)
Supplement: Supplementary file 1 [file genes-16-00293-s001.zip › Supplementary Figure s1.pdf]

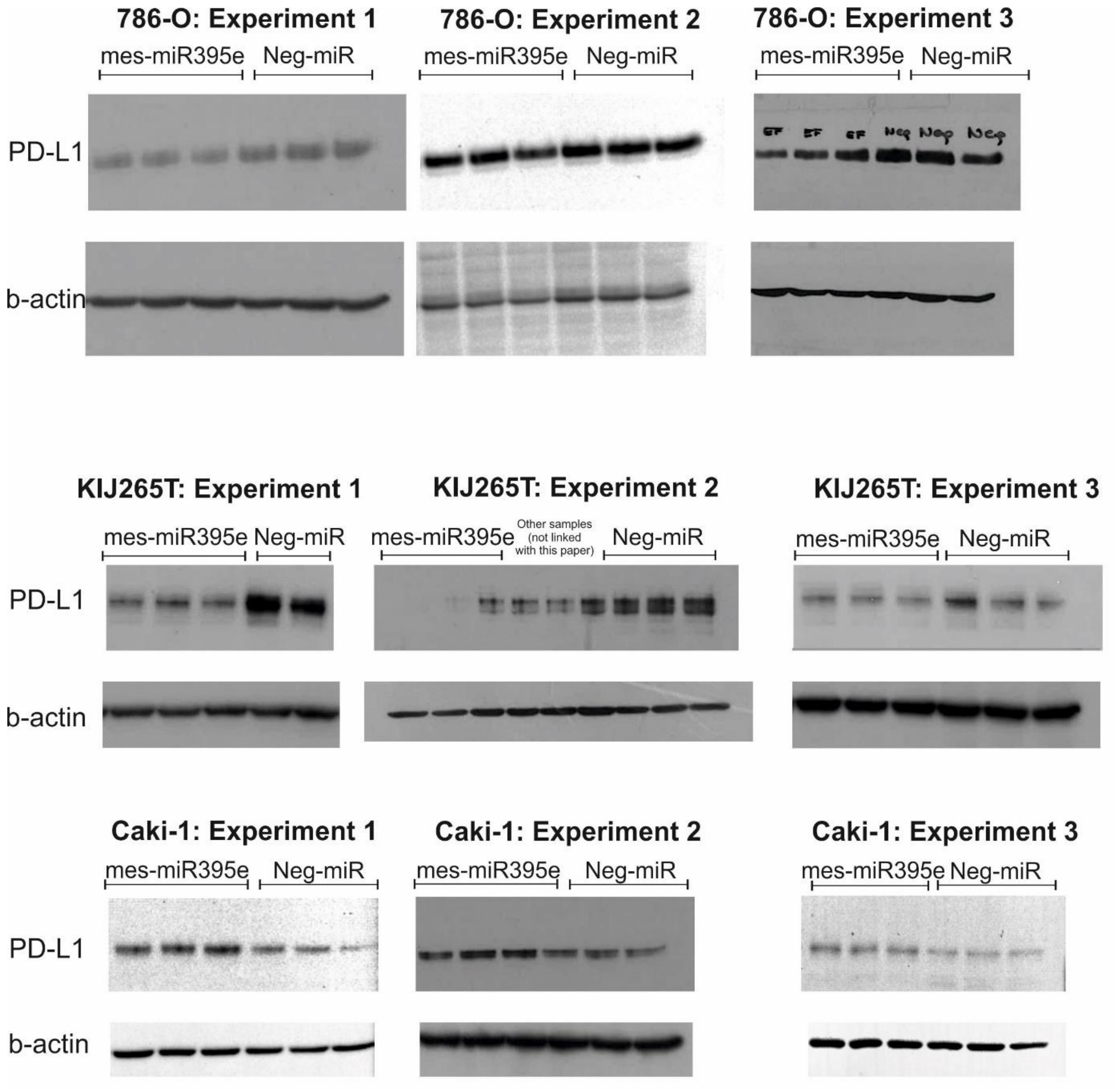

Supplementary Figure S1. Full data of Western blot analysis of RCC cell lines transfected with mes-miR395e mimic or non-targeting scrambled control oligonucleotide.
