# Supplementary material for: miR395e from Manihot esculenta Decreases Expression of PD-L1 in Renal Cancer: A Preliminary Study
Source: Genes (Basel). 2025 Feb 27;16(3):293. doi: 10.3390/genes16030293 (PMC11942022; doi:10.3390/genes16030293)
Supplement: Supplementary file 1 [file genes-16-00293-s001.zip › Supplementary Figure s2.pdf]

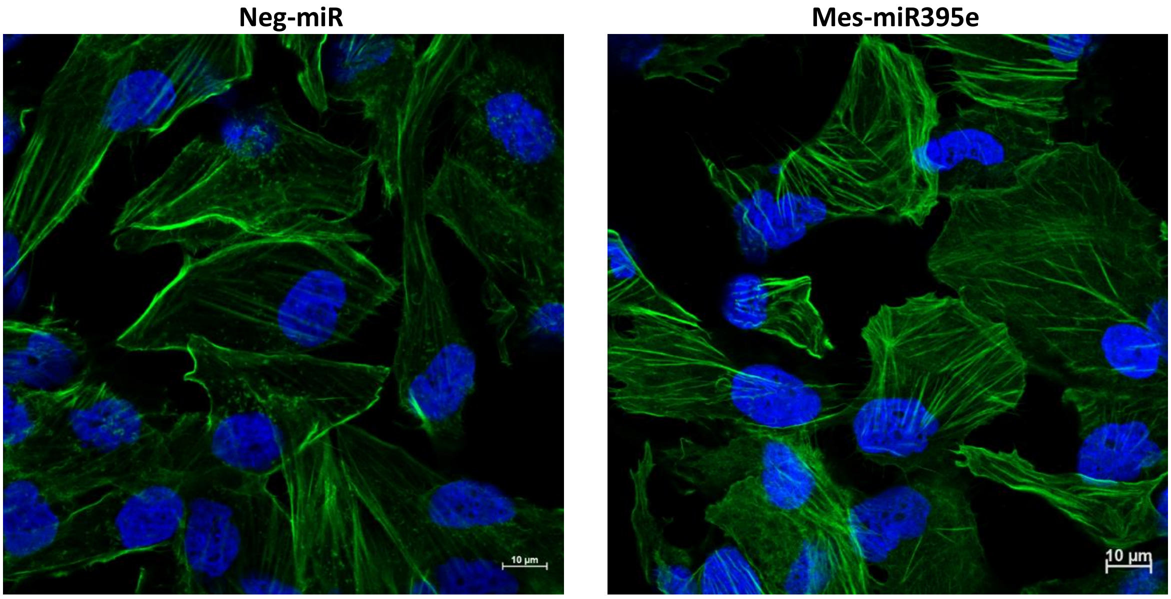

Supplementary Figure S2. Representative confocal microscopy images of 786-O cells transfected with non-targeting scrambled control oligonucleotide (Neg-miR) or mes-miR395e mimic.
